# A Rare Case of Rosai Dorfman Destombes Disease Presenting With Lupus Nephritis and Central Nervous System Small Vessel Vasculitis

**DOI:** 10.7759/cureus.72684

**Published:** 2024-10-30

**Authors:** Sharon Kandari, Anshuman Biswal, Rohit Puri, Prateek Kumar Panda, Arvind Kumar

**Affiliations:** 1 Nephrology, All India Institute of Medical Sciences, Rishikesh, Rishikesh, IND; 2 Paediatrics, All India Institute of Medical Sciences, Rishikesh, Rishikesh, IND; 3 Pathology and Laboratory Medicine, All India Institute of Medical Sciences, Rishikesh, Rishikesh, IND

**Keywords:** rdd, rosai-dorfman disease. sinus histiocytosis with massive lymphadenopathy. destombes–rosai–dorfman disease. spine. magnetic resonance imaging. computed tomography, sle and lupus nephritis, small vessel vasculitis, systemic steroids

## Abstract

Rosai-Dorfman-Destombes (RDD) disease is a rare syndrome characterised by benign lymphoproliferative disorder with sinus histiocytosis that presents with massive lymphadenopathy. It occurs mainly in children and young adults. It can be associated with autoimmune diseases like systemic lupus erythematosus (SLE). We present a 15-year-old male case of RDD who was diagnosed with SLE with class IV lupus nephritis and central nervous system small vessel vasculitis. Sensorium improved and lymph node size decreased with steroids. Hence, SLE can present with nephrito-nephrotic syndrome and central nervous system (CNS) small vessel vasculitis without any other systemic involvement. Steroids can be helpful in the management of RDD. Therefore, we should consider the possibility of RDD in cases of SLE with generalised lymphadenopathy, even though the association is rare.

## Introduction

Rosai-Dorfman-Destombes (RDD) disease is a rare non-Langerhans cell histiocytosis characterised by tissue accumulation of histiocytes. RDD is a mostly self-limited disorder with unknown aetiology [1]. In 1965, Pierre Paul Louis Lucien Destombes first reported four children and young adults with lymphadenopathy and sinus histiocytosis [2]. It has a prevalence of 1:200,000 in the US [3]. Around 100 new cases per year are detected with RDD in the United States [3]. Children and young adults commonly experience it. In RDD, histiocytes are S100+, CD68+, and CD1a− and demonstrate emperipolesis [1]. RDD can occur as an isolated disorder or in association with autoimmune and malignant diseases. Around 10% of cases of RDD coexist with immunologic diseases. These include idiopathic juvenile arthritis, autoimmune haemolytic anaemia, and systemic lupus erythematosus (SLE) [4]. Steroids, immunomodulators, and surgical excision can be used in the management of RDD. We hereby present a rare case of lymphadenopathy due to RDD disease, who was diagnosed with SLE with lupus nephritis and central nervous system (CNS) small vessel vasculitis.

## Case presentation

**Figure 1:**
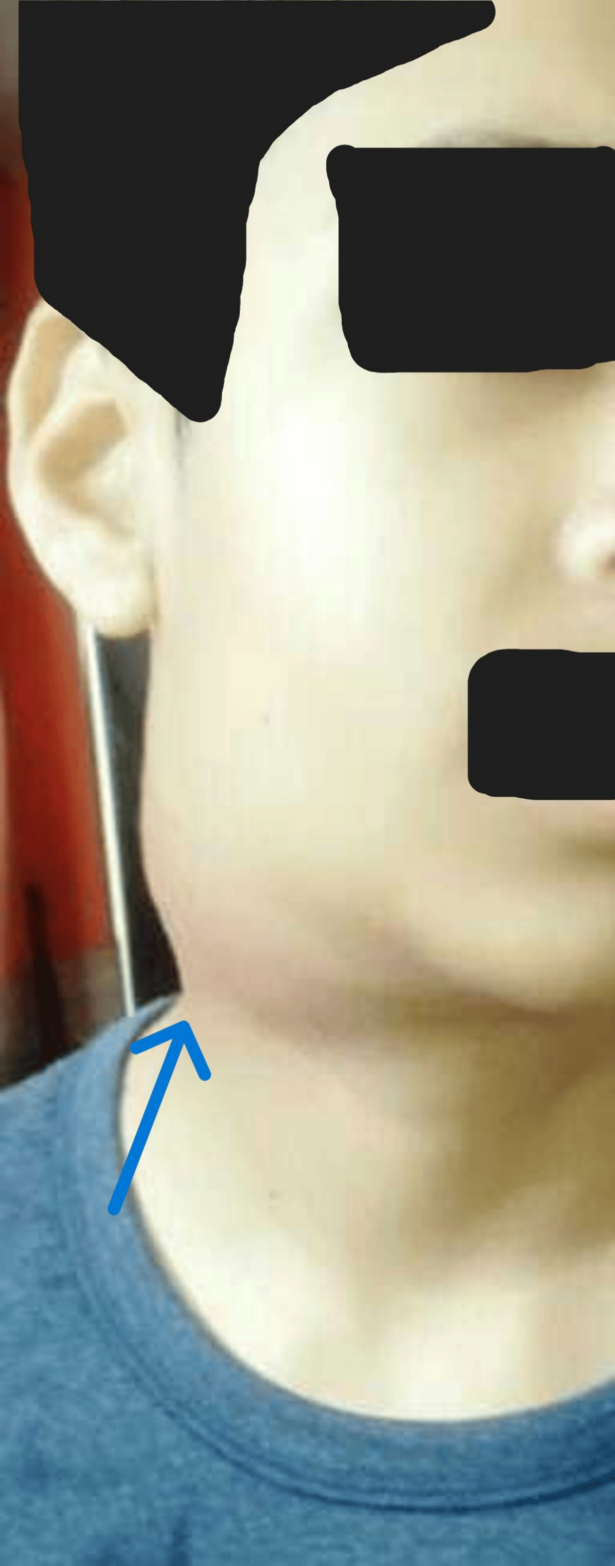
Enlarged right submandibular and cervical lymph node

**Figure 2:**
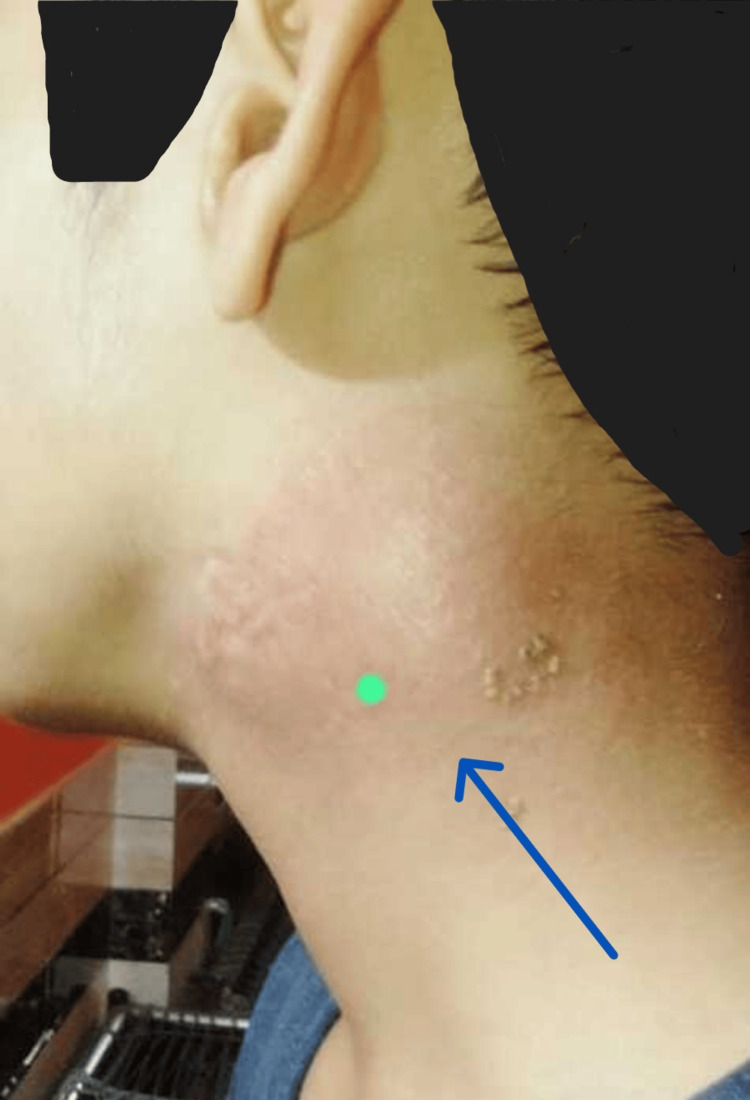
Enlarged left cervical lymph node

**Figure 3:**
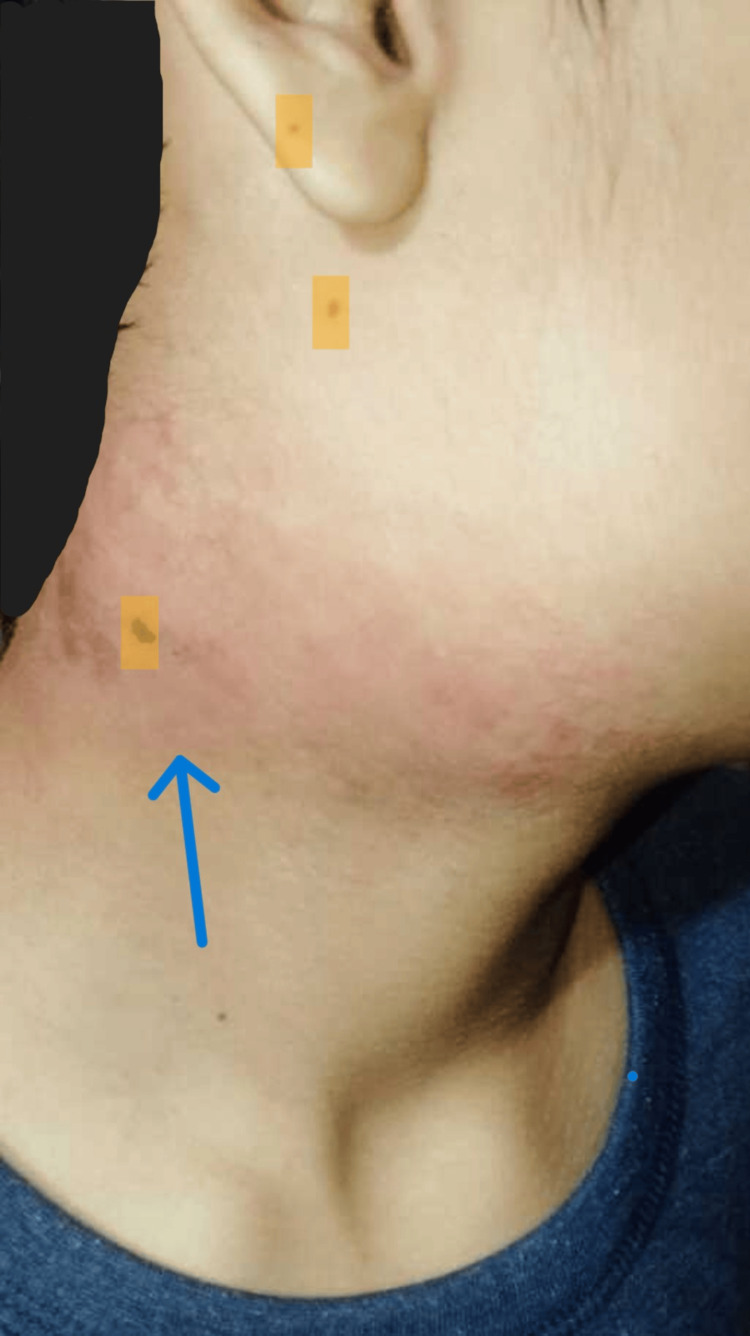
Enlarged right cervical lymph node

**Figure 4:**
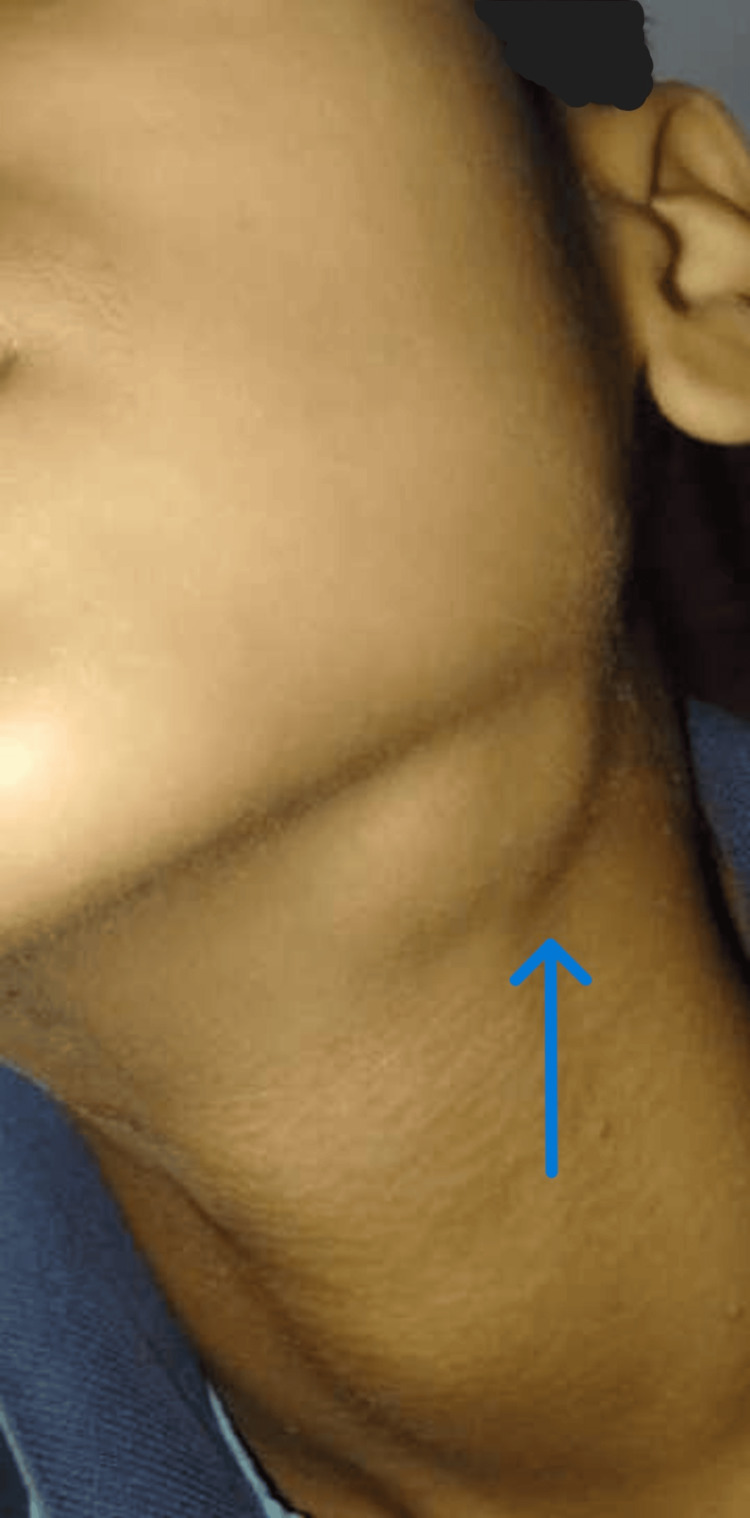
Enlarged left submandibular lymph node

A 15-year-old male developed left submandibular swelling in November 2022. The swelling was firm and non-painful, with no superficial skin discolouration or discharge. There was no history of fever, cough, night sweats, or weight loss. There was no contact history for Koch’s disease. Ultrasonography (USG) of the neck done outside showed enlarged lymph nodes of size 15-25 mm with well-defined margins and internal cystic areas in the left submandibular region. He was given oral antibiotics. Swelling persisted. In June 2023, a left submandibular salivary gland biopsy was done outside. It showed salivary gland acini with periductal sclerosis, acinar atrophy, and infiltration by lymphoplasmacytic infiltrate, suggestive of chronic sclerosing sialadenitis. It showed reactive lymph nodes with no evidence of granuloma, necrosis, or malignancy. He developed similar swellings in the right submandibular and bilateral inguinal areas over the next six months. He experienced facial puffiness and bilateral lower limb swelling in January 2024. He experienced a decrease in his urine output and the presence of froth in it. There was no history of chest pain, palpitations, yellowish discolouration of skin, sclera, or shortness of breath. There was no complaint of a sore throat or passing of cola-coloured urine. There was no history of alopecia, minor joint pain, rash, or recurrent oral ulcers. He had altered behaviour, decreased sleepiness, and irrelevant talks since February 2024. There was no history of headache, blurring of vision, fever, loose stools, vomiting, or limb weakness. With these complaints, he presented to Nephrology OPD AIIMS, Rishikesh, on February 26, 2024. Enlargement of both right and left submandibular and cervical lymph nodes is depicted in Figures 1-4.

The blood pressure of the patient was 160/90 mm hg. We started him on the oral tablet Amlodipine 5 mg once daily. The urine dipstick revealed a protein level of 4+ and a blood level of 2+. Viral markers were non-reactive. Table 1 shows investigations at the time of presentation.

**Table 1:** Table showing the existence of anaemia, hypoalbuminemia and proteinuria

Parameters	Patient values	Reference range
Haemoglobin	8.69 g/dL	12–15 g/dL
TLC	9.1 x 10^3^/μL	4–11 x 10^3^/μL
Platelet count	157 x 10^3^/μL	150–400 x 10^3^/μL
Urea	65 mg/dL	17–43 mg/dL
Creatinine	1.2 mg/dL	0.55–1.02 mg/dL
Serum albumin	2.4 gm/dL	3.5–5.2 g/dL
Serum cholesterol	170 mg/dL	123–200 mg/dL
Serum triglyceride	132 mg/dL	50–150 mg/dL
24-hour urine protein	6.51 g/day	0.04–0.15 g/day

**Figure 5:**
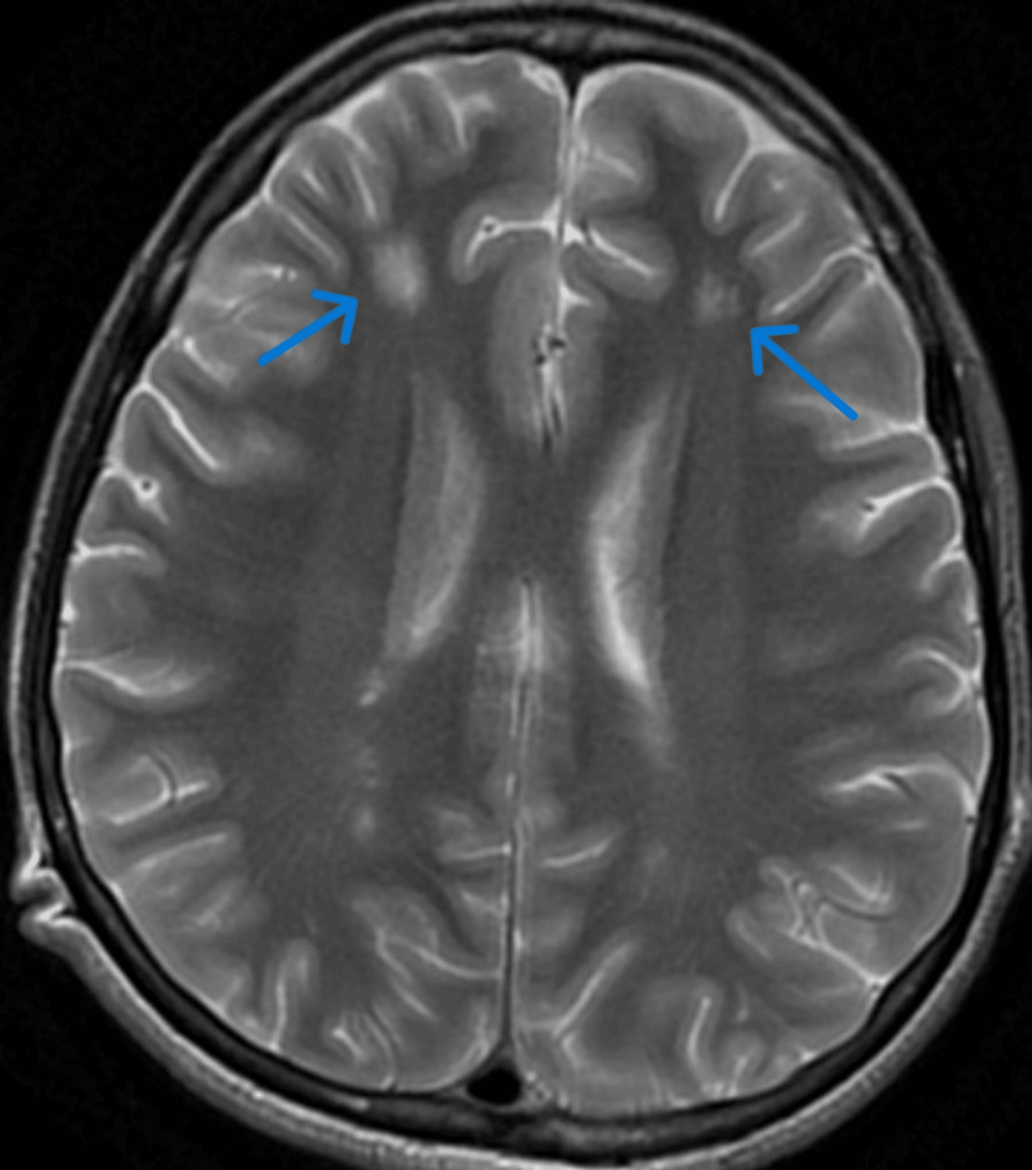
Contrast-enhanced T2-weighted MRI brain film showing frontal hyperintensities

**Figure 6:**
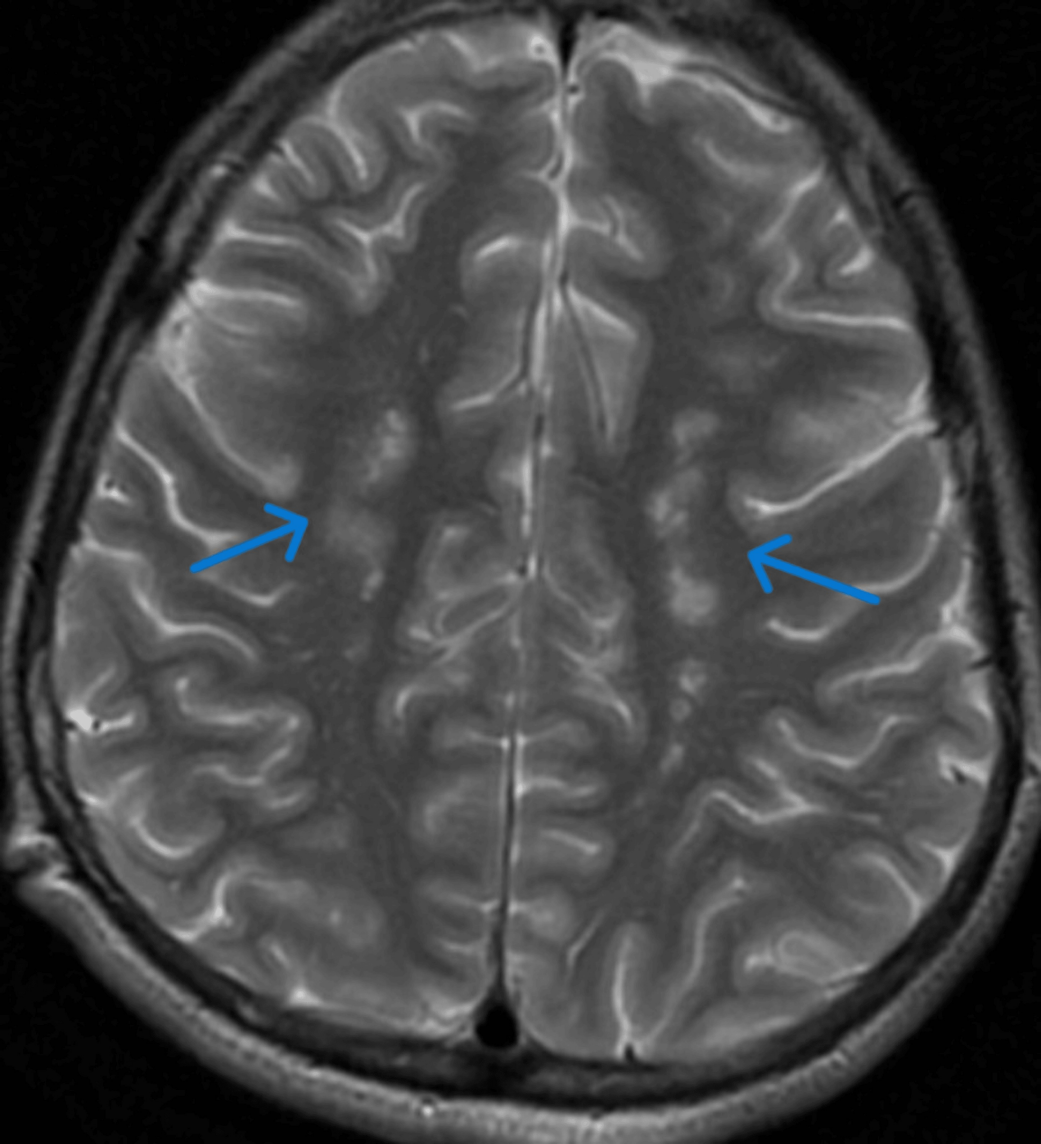
Contrast-enhanced T2-weighted MRI brain film showing parieto-temporal hyperintensities

He was diagnosed with the nephrito-nephrotic syndrome. He was planned for a renal biopsy. Serum electrolytes and sugar levels were within normal limits. Cerebrospinal fluid (CSF) analysis was acellular. The CSF cartridge-based nucleic acid amplification test (CB-NAAT) for acid-fast bacilli was negative, and the aerobic culture was sterile. Contrast-enhanced MRI brain showed patchy areas of multiple hyperintensities in subcortical white matter in bilateral frontal and parietal lobes with diffusion restriction, with a punctate area of multiple blooming in the frontotemporal parietal area suggestive of microbleeds with the impression of small vessel vasculitis. The contrast-enhanced images of the patient's MRI brain can be found in Figures 5-6.

**Figure 7:**
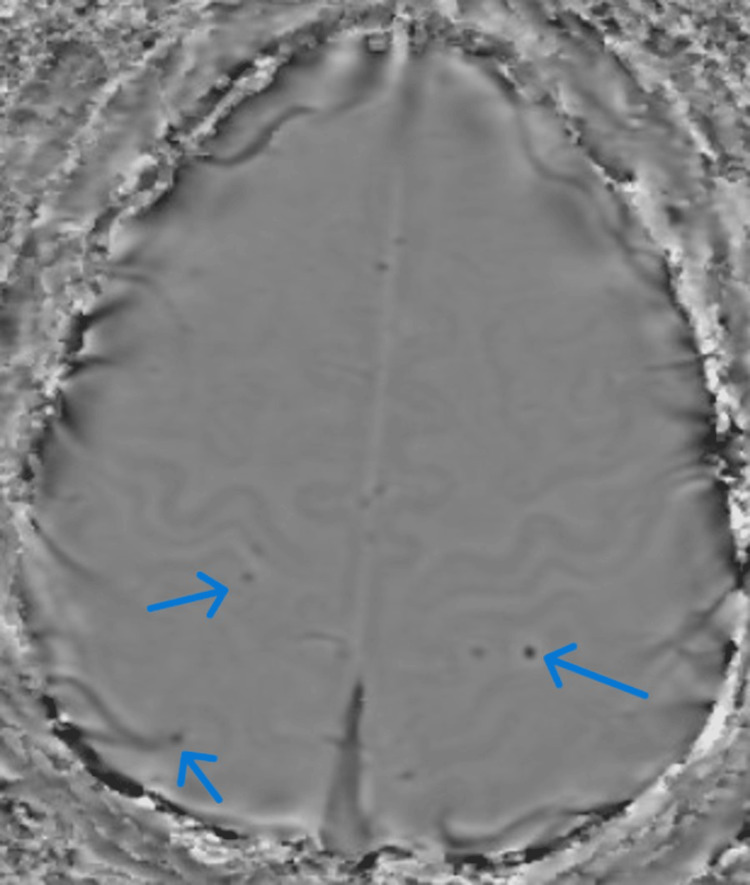
Contrast-enhanced MRI brain film showing blooming at parieto-temporal areas suggestive of microbleeds

Figure 7 shows contrast-enhanced MRI images of blooming at parietal and temporal areas of the brain suggestive of microbleeds.

**Figure 8:**
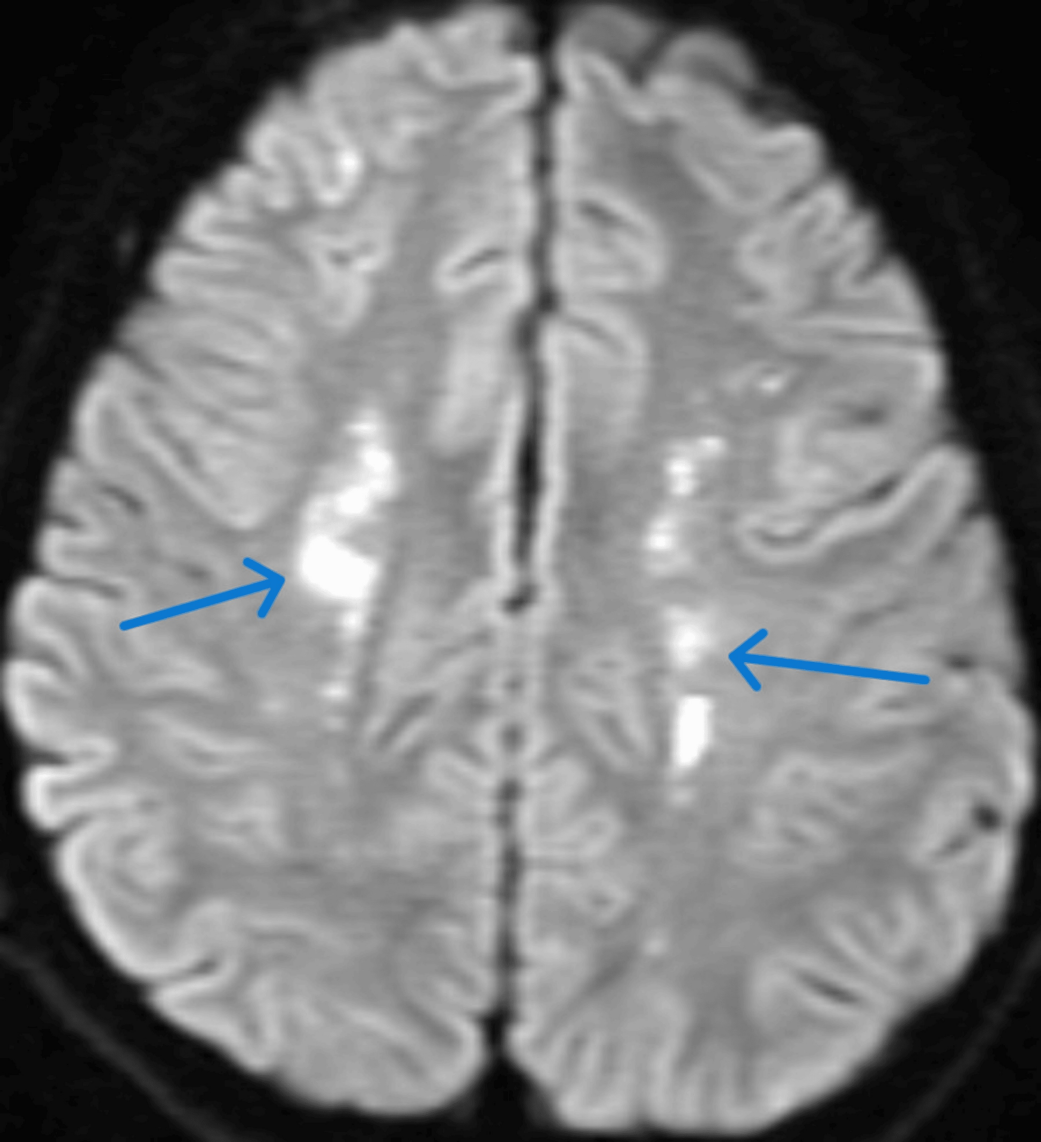
Contrast-enhanced MRI brain DWI sequence showing diffusion restriction at parieto-temporal areas suggestive of small vessel vasculitis

Figure 8 shows a contrast-enhanced MRI DWI sequence image of diffusion restriction suggestive of small vessel vasculitis.

**Figure 9:**
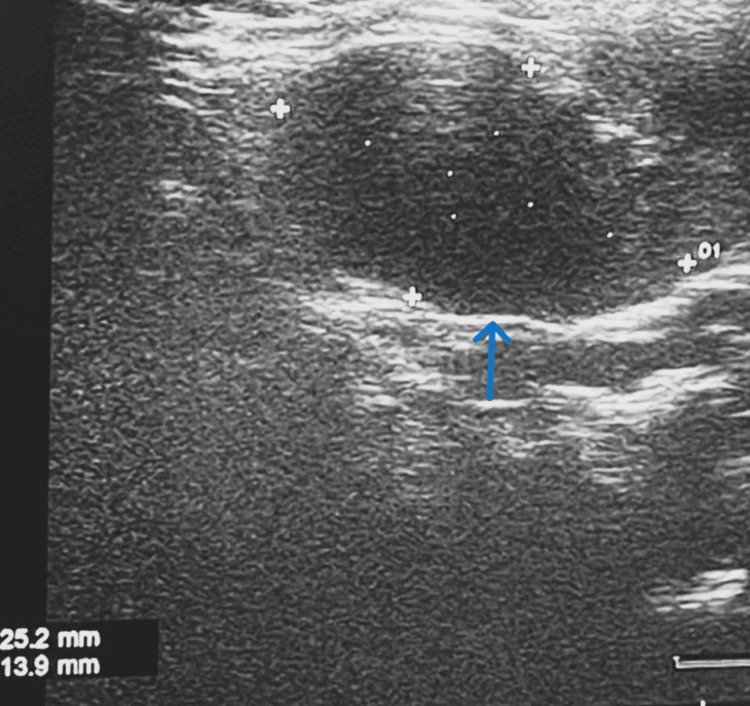
USG neck showing enlarged right cervical lymph node (2.5 cm × 1.3 cm)

**Figure 10:**
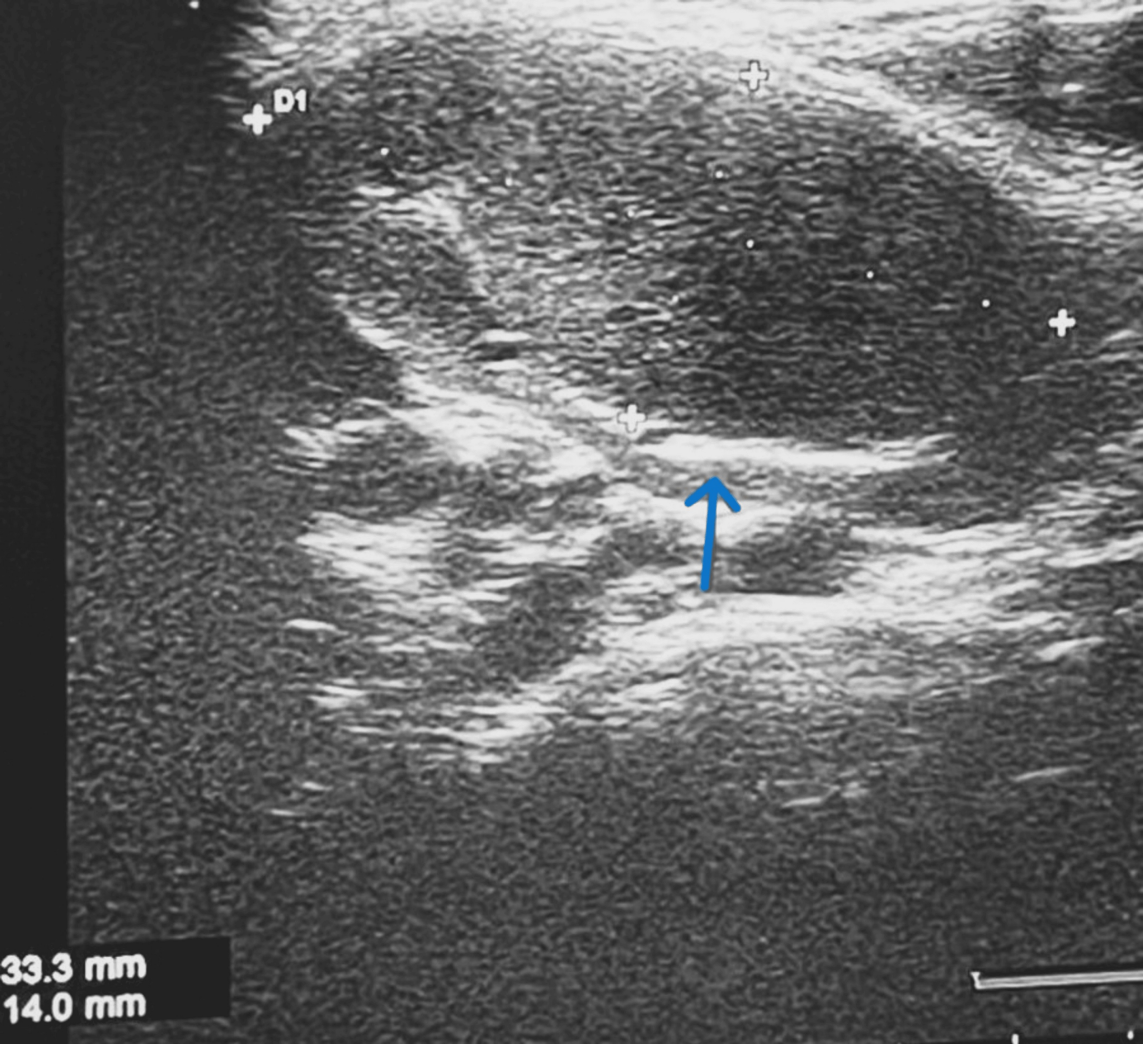
USG neck showing enlarged left cervical lymph node (3.3 cm × 1.4 cm)

He was diagnosed with CNS vasculitis due to lupus. USG neck showed bilaterally enlarged cervical lymph nodes in levels II, III, and IV, as well as enlarged submandibular and inguinal lymph nodes. Figures 9-10 show USG images of the enlarged right cervical (2.5 cm × 1.3 cm) and left cervical lymph node (3.3 cm × 1.4 cm), respectively.

**Figure 11:**
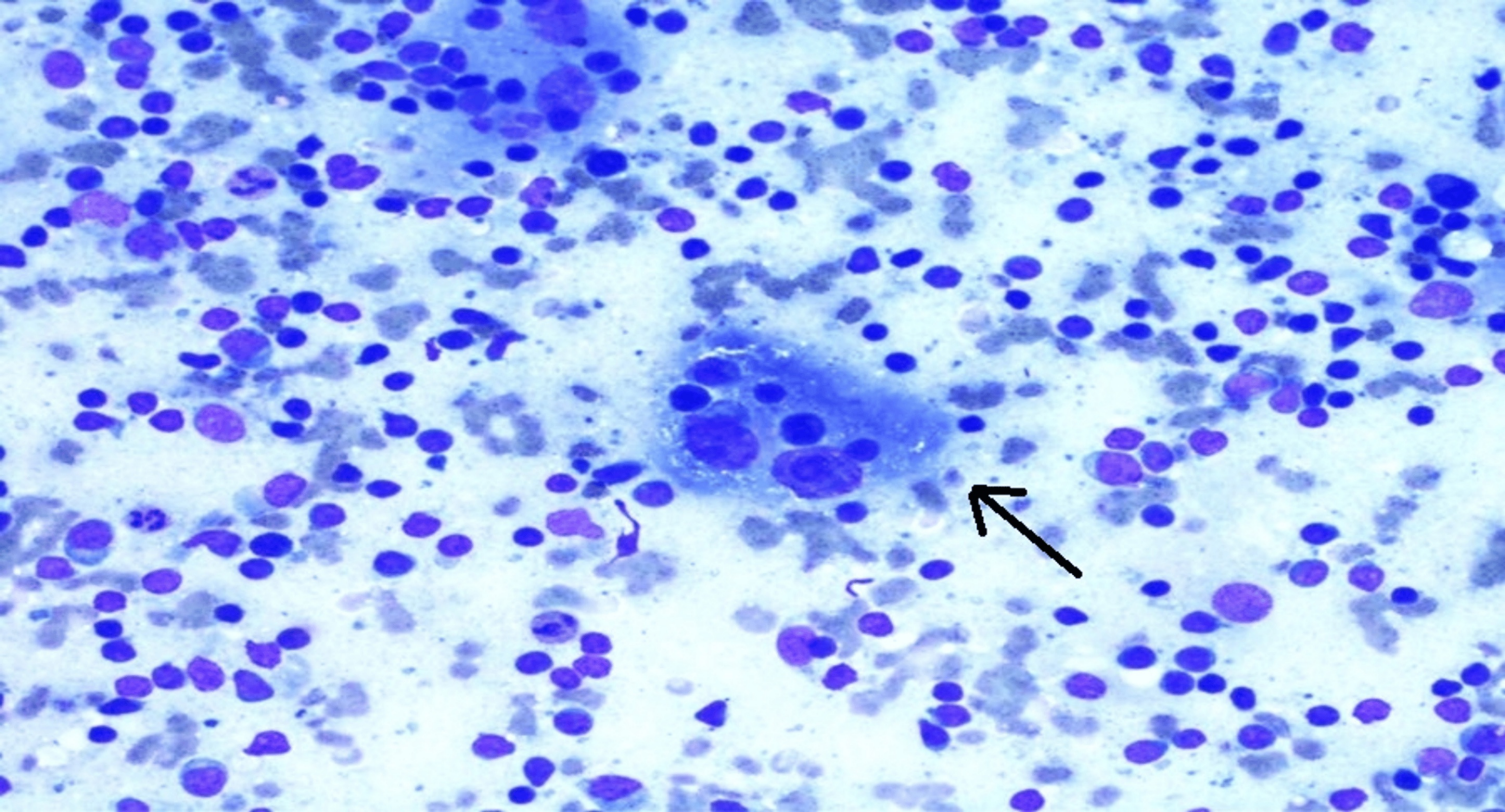
Fine needle aspiration cytology of cervical lymph node showing predominance of histiocytes It shows histocytes having a large vesicular nucleus with engulfed intact inflammatory cells in abundant foamy vacuolated cytoplasm. This process is called emperipolesis. Giemsa stain, 400×.

Fine needle aspiration cytology (FNAC) of the cervical lymph node showed a polymorphous population of lymphoid cells showing emperipolesis with predominant histiocytes with no granuloma or features for malignancy suggestive of benign histiocytosis, i.e., RDD disease. Figure 11 shows a FNAC cervical lymph node.

**Figure 12:**
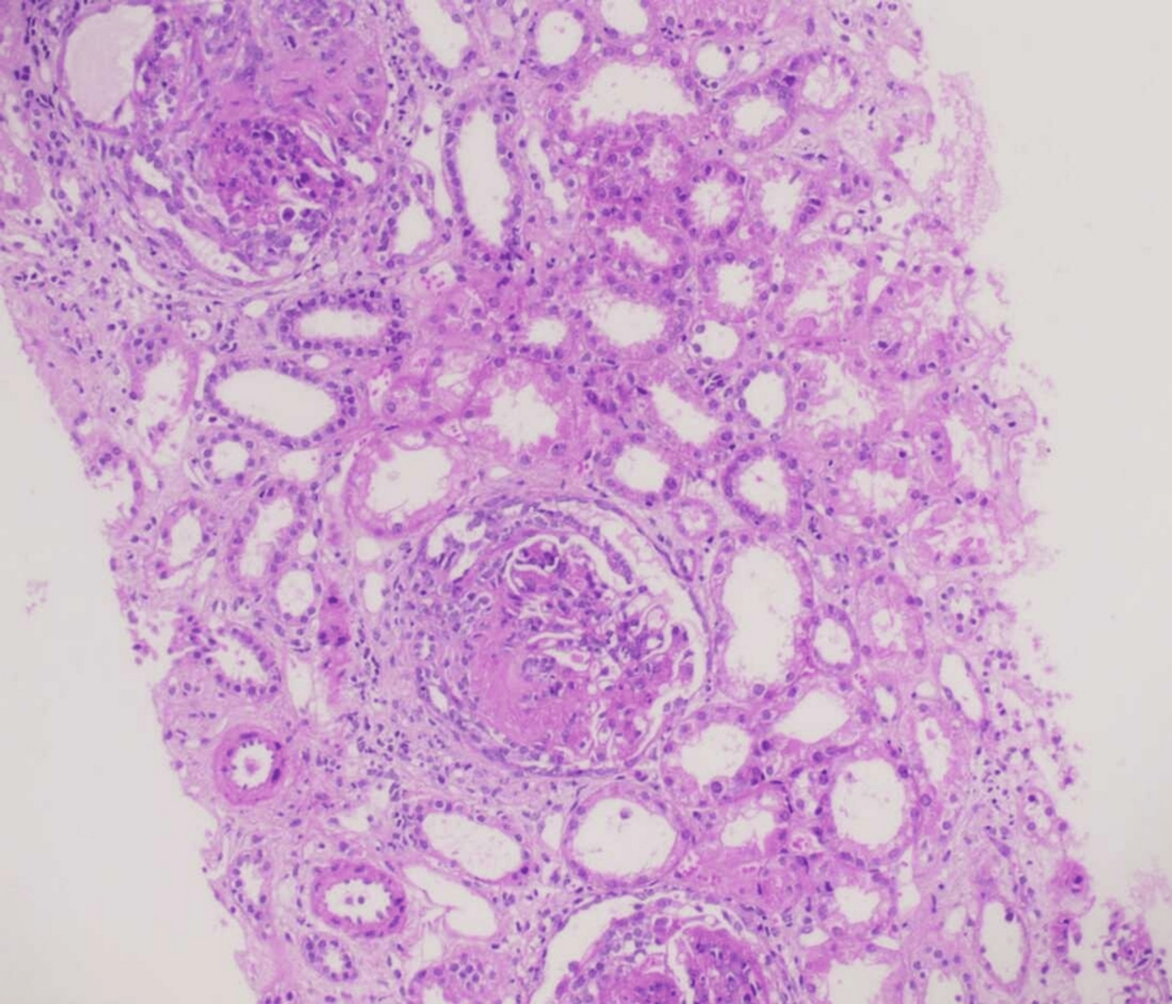
Light microscopy of renal biopsy showing mesangial and endocapillary hypercellularity There is the presence of cellular crescents and segmental fibrinoid tuft necrosis suggestive of proliferative morphology. H&E stain, 200×.

The anti-nuclear antibody immunofluorescence assay was 3+ positive with end titers of 1:1000 (primary dilution titer 1:100). The ANA pattern was nuclear homogenous (2+ for Smith, nucleosomes, histones, and U1 RNP). Serum C3 was 61.8 (low) and C4 was <8 (low). He was given pulse methylprednisolone 500 mg for three consecutive days, followed by oral steroids (1 mg/kg/day) - prednisolone 40 mg for the first two weeks for possible lupus nephritis and CNS lupus vasculitis. A gradual improvement in sensorium was observed over a period of the next seven days. USG kidneys, ureters, and bladder showed right kidney - 10.9 CM and left kidney - 11 cm with normal echogenicity and maintained cortico-medullary differentiation. A renal biopsy was done, which showed 14 glomeruli, and none were globally sclerosed. There was mild to moderate mesangial hypercellularity, along with matrix expansion and endocapillary hypercellularity. Ten glomeruli had cellular crescents, with three glomeruli showing segmental fibrinoid tuft necrosis. Figure 12 showed proliferative morphology in renal biopsy.

Interstitial fibrosis and tubular atrophy were <10%. Immunofluorescence showed a full-house pattern. The activity score was 15/24, and the chronicity score was 2/12. The renal biopsy report suggested diffuse proliferative lupus nephritis (class IV lupus nephritis) as per ISN/RPS (2018). The patient was diagnosed with SLE with lupus nephritis and CNS lupus vasculitis as per ACR/EULAR criteria (2019). He was given an injection of cyclophosphamide 750 mg according to the NIH regimen as per his clinical profile. Lymph node size decreased within two weeks of the start of steroids. He achieved partial renal remission by the sixth dose of cyclophosphamide. Twenty-four-hour urine protein was 3.1 g/day before the sixth dose of cyclophosphamide. The steroid dose was tapered. Repeat USG of the neck and inguinal area showed no lymphadenopathy. He has completed six doses of cyclophosphamide injection, 750 mg. He was started on mycophenolate mofetil 500 mg twice daily as a maintenance agent for lupus.

## Discussion

Rosai-Dorfman-Destombes disease is a rare disease characterised by benign lymphoproliferative disorder with sinus histiocytosis that presents with massive lymphadenopathy. Classical RDD presents with bilateral cervical lymphadenopathy. Extra-nodal disease is seen in 43% of patients with RDD [1]. RDD is more common in males [5]. Around 10% of cases of RDD can have an association with autoimmune diseases, i.e., SLE. In the Indian scenario, there is the possibility of disseminated Koch’s, lymphoproliferative disorder, and malignancy with metastases, which can lead to generalised lymphadenopathy. Renal involvement in SLE includes proteinuria >0.5 g/24-hour, haematuria, RBC cast in urine, and features suggestive of lupus nephritis in renal biopsy as per ACR/EULAR criteria (2019). CNS involvement includes presentation as delirium, psychosis, and seizure as per ACR/EULAR criteria (2019). Around 5% of cases of RDD can have CNS involvement (75% as intracranial and 25% as spinal lesions). There may be headaches, seizures, cranial nerve deficits, and motor or sensory abnormalities as a part of CNS involvement in RDD [5].

There was no history of low-grade fever, weight loss, or night sweats. FNAC of the lymph node in our case did not show evidence of Reed Sternberg cells found in Hodgkin’s lymphoma. CB NAAT for AFB was negative. There were no granulomas or caseating necrosis in FNAC. FNAC showed emperipolesis with predominant histiocytes suggestive of RDD. Due to financial constraints, S100, CD68, CD1a, and CD207 could not be done. Immunohistochemistry and excisional biopsy were not done in our case, which are limitations in our investigative workup.

Approximately 50% of SLE patients can have kidney involvement [6]. Class IV lupus nephritis is the most common histopathological finding [7]. About 25 to 75% of patients with SLE can have neurological manifestations [8]. Involvement of the musculoskeletal system is widespread in SLE [9]. Around 95% of patients with SLE have arthralgia and arthritis [10]. About 70-80% of patients develop skin lesions during disease. Skin lesions can be an initial presentation in approximately 20-25% of cases [11]. In our case, the patient presented with nephrito-nephrotic syndrome and had CNS small vessel vasculitis without any dermatological, musculoskeletal, or other systemic manifestations, which is rare.

Kaur et al. reported a 37-year-old man with a history of SLE developed a tender lump over his right upper eyelid. Microscopic examination and IHC were suggestive of RDD. There was axillary, mesenteric, and iliac lymphadenopathy in the CT scan. There was spontaneous resolution of nodular lesions and lymph nodes in CT [12]. Amoako et al. reported a case of a 13-year-old male with progressive left-sided neck swelling. He had an enlarged left cervical and supraclavicular lymph node. FNAC and IHC were suggestive of RDD. Cervical lymph node size and pain over the lymph node decreased following the start of steroids [13]. There is spontaneous remission in about 20-50% of cases of RDD. Systemic corticosteroids and surgical excision are commonly used as first-line treatments. Fifty-six percent of the cases responded to steroids in a study done by Goyal et al. [14]. In our case, there was the resolution of lymphadenopathy with steroids within two weeks of the start of therapy. Hence, steroids can play an important role in the management of RDD presenting with lymphadenopathy.

## Conclusions

We should consider the possibility of RDD when there is generalised lymphadenopathy in patients with SLE, although the association is rare. SLE can present as nephrito-nephritic syndrome and CNS small vessel vasculitis only without any other systemic involvement. Steroids can be helpful in managing lymphadenopathy in RDD.
